# STAT and Janus kinase targeting by human herpesvirus 8 interferon regulatory factor in the suppression of type-I interferon signaling

**DOI:** 10.1371/journal.ppat.1010676

**Published:** 2022-07-01

**Authors:** Qiwang Xiang, Zunlin Yang, John Nicholas

**Affiliations:** Sidney Kimmel Comprehensive Cancer Center at Johns Hopkins, Department of Oncology, Johns Hopkins University School of Medicine, Baltimore, Maryland, United States of America; Hannover Medical School, GERMANY

## Abstract

Human herpesvirus 8 (HHV-8), also known as Kaposi’s sarcoma (KS)-associated herpesvirus, is involved etiologically in AIDS-associated KS, primary effusion lymphoma (PEL), and multicentric Castleman’s disease, in which both viral latent and lytic functions are important. HHV-8 encodes four viral interferon regulatory factors (vIRFs) that are believed to contribute to viral latency (in PEL cells, at least) and/or to productive replication via suppression of cellular antiviral and stress signaling. Here, we identify vIRF-1 interactions with signal transducer and activator of transcription (STAT) factors 1 and 2, interferon (IFN)-stimulated gene factor 3 (ISGF3) cofactor IRF9, and associated signal transducing Janus kinases JAK1 and TYK2. In naturally infected PEL cells and in iSLK epithelial cells infected experimentally with genetically engineered HHV-8, vIRF-1 depletion or ablation, respectively, led to increased levels of active (phosphorylated) STAT1 and STAT2 in IFNβ-treated, and untreated, cells during lytic replication and to associated cellular-gene induction. In transfected 293T cells, used for mechanistic studies, suppression by vIRF-1 of IFNβ-induced phospho-STAT1 (pSTAT1) was found to be highly dependent on STAT2, indicating vIRF-1-mediated inhibition and/or dissociation of ISGF3-complexing, resulting in susceptibility of pSTAT1 to inactivating dephosphorylation. Indeed, coprecipitation experiments involving targeted precipitation of ISGF3 components identified suppression of mutual interactions by vIRF-1. In contrast, suppression of IFNβ-induced pSTAT2 was effected by regulation of STAT2 activation, likely via detected inhibition of TYK2 and its interactions with STAT2 and IFN type-I receptor (IFNAR). Our identified vIRF-1 interactions with IFN-signaling mediators STATs 1 and 2, co-interacting ISGF3 component IRF9, and STAT-activating TYK2 and the suppression of IFN signaling via ISGF3, TYK2-STAT2 and TYK2-IFNAR disruption and TYK2 inhibition represent novel mechanisms of vIRF function and HHV-8 evasion from host-cell defenses.

## Author summary

Viral interferon regulatory factors (vIRFs) encoded by Kaposi’s sarcoma- and lymphoma-associated human herpesvirus 8 (HHV-8) are mediators of protection from cellular antiviral responses and therefore are considered to be pivotal for successful *de novo* infection, latency establishment and maintenance, and productive (lytic) replication. Identification and characterization of their interactions with cellular proteins, the functional consequences of these interactions, and the operation of these mechanisms in the context of infection has the potential to enable the development of novel antiviral strategies targeted to these interactions and mechanisms. In this report we identify vIRF-1 interactions with transcription factors STAT1 and STAT2, the co-interacting component, IRF9, of the antiviral interferon (IFN)-induced transcription complex ISGF3, and the ability of vIRF-1 to inhibit activation and functional associations of IFN-I receptor- and STAT1/2-kinase TYK2, suppress STAT1/2 activation, and dissociate STAT1 from IFN-induced ISGF3 to blunt IFN signaling and promote STAT1 inactivation. These interactions and activities, which mediate suppression of innate cellular defenses against virus replication, represent novel properties among vIRFs and could potentially be exploited for antiviral and therapeutic purposes.

## Introduction

AIDS-associated Kaposi’s sarcoma-, primary effusion lymphoma (PEL)-, and multicentric Castleman’s disease-associated human herpesvirus 8 (HHV-8) contains certain genes, including those encoding four viral interferon regulatory factors (vIRFs), that are particular to this and closely related macaque gammaherpesviruses [1–5]. Each HHV-8 vIRF is known to have a particular cohort of cellular protein targets, which are generally inhibited by vIRF binding and comprise mediators of innate immune and stress signaling [6,7]. Therefore, it is speculated that the vIRFs serve to blunt cellular signaling pathways that are not conducive to successful virus infection and replication. Nonetheless, the actual functions of the vIRFs and particular vIRF-protein interactions in HHV-8 *de novo* infection, latency, and productive replication are still poorly understood. However, it is known that in PEL cells, vIRFs 1, 2 and 3, which are expressed at least to some degree in the latent phase, support PEL cell viability, and in lytic replication, vIRF-1 has been shown to promote virus production from PEL cells and iSLK epithelial cells (inducible for immediate-early RTA expression and used experimentally to host genetically modified HHV-8) and viral lytic gene expression in the former [8–11]. Counterintuitively, vIRF-2 and vIRF-3 inhibit productive replication in PEL and iSLK cells, as does vIRF-2 in HuARLT endothelial cells [9,10,12]. Indeed, identified activities of vIRF-2 include the activation of innate immune signaling leading to interferon induction, direct induction of interferon-stimulated genes, and specific repression of early viral genes [10,12]. The combined effects and particular roles of the vIRFs in the context of overall HHV-8 biology *in vivo* are unknown, but it is notable that the vIRFs in HHV-8-related rhesus rhadinovirus are, together, necessary for suppression of innate and adaptive immune responses to virus infection and for establishment of normal latent viral loads in infected macaques [13].

HHV-8 vIRF-1 has been reported to interact in inhibitory fashion with IRF-1, IRF-associating CBP/p300 transcriptional activators, innate-immune signaling protein MAVS, and pro-apoptotic BH3-only proteins (BOPs), GRIM19, and Smad3/4 transcription factors [14–20]; additionally, it has been demonstrated that vIRF-1 is a strong suppressor of viral DNA-activated cGAS-STING antiviral signaling, via STING-TBK1 disruption [21]. The deubiquitinase USP7 is also targeted by vIRF-1, as it is by the other HHV-8 vIRFs [9,10,22,23]. Although genetic analyses have demonstrated the importance of vIRF-1 interactions with BOPs and USP7 for efficient productive replication in endothelial cells and in PEL and iSLK cells, respectively [9,17,24], the roles of vIRF-1, and other vIRF, interactions in virus biology remain largely undetermined. Identification and characterization of the full repertoire of interactions is necessary to elucidate the mechanisms of vIRF functions and identify those interactions that may represent appropriate drug targets. Liberating the infected cell from inhibitory vIRF interactions with mediators of host-cell defense, individually or in combination, would be expected to effectively block virus productive replication.

Interferon signaling represents a major mechanism of cellular defense against virus infection. Interferon type-I (α/β) signal transduction, involving IFN receptor-activated STAT1 and STAT2 and their association with IRF9, comprises the second wave of innate immune signaling, following microbial pattern recognition receptor-mediated IRF activation and IFN-I gene induction [25,26]. IFN-I activation of IFNα/β receptors 1 and 2 (IFNAR1, IFNAR2), which form heterodimers upon IFN-I binding, leads to auto/cross-phosphorylation of the IFNAR-bound Janus kinases JAK1 and TYK2 and IFNAR1/2, then to recruitment and tyrosine-phosphorylation (at residues 701 and 690, respectively) of STAT1 and STAT2 and associated STAT SH2 domain-mediated homo- (STAT2_2_) or hetero-dimerization in association with STAT2-interacting IRF9. These activated STAT complexes enter the nucleus and induce the expression of IFN-stimulated genes (ISGs). STAT1:STAT2:IRF9 (ISGF3) and STAT2_2_:IRF9 complexes bind to particular promoter *cis*-elements conforming to a consensus sequence known as the IFN-stimulated response element (ISRE) [27–29]. STAT1 homodimers, activated also (and mainly) by IFNγ, target IFNγ-activated promoter elements (γ-activated sequences, GAS) to stimulate a distinct, but overlapping, set of ISGs [30,31]. In addition to induction by IFN-I, the ISGF3 complex can be induced also by IFNα/β-downstream IFNγ- and IFNλ-activated signaling via IFNGR1/2 and IFNLR1/IL-10Rβ receptors, respectively [32]. The ISGF3 complex in addition to STAT1_2_ homodimers are major mediators of the immediate innate immune response to virus and other microbial infections. HHV-8 vIRF-1 has been reported to inhibit signaling by IFN-I (α/β) and IFNγ [11,33,34], but the mechanisms underlying these activities are unknown.

Here, we identify HHV-8 vIRF-1 interactions with STAT1 and STAT2, in addition to associated IRF9, and suppression of related IFN-I (IFNβ) signal transduction in both transfected and HHV-8-infected cells, and also identify inhibitory interaction of vIRF-1 with IFN-I-receptor-associating and STAT1/2-activating kinase TYK2. Interactions of vIRFs with STATs, IRF9, and Janus kinases have not previously been reported; they are likely to represent important mechanisms of HHV-8 innate-immune evasion required for successful infection and replication.

## Results

### HHV-8 vIRF-1 interactions with ISGF3 components STAT1, STAT2 and IRF9

**Fig 1:**
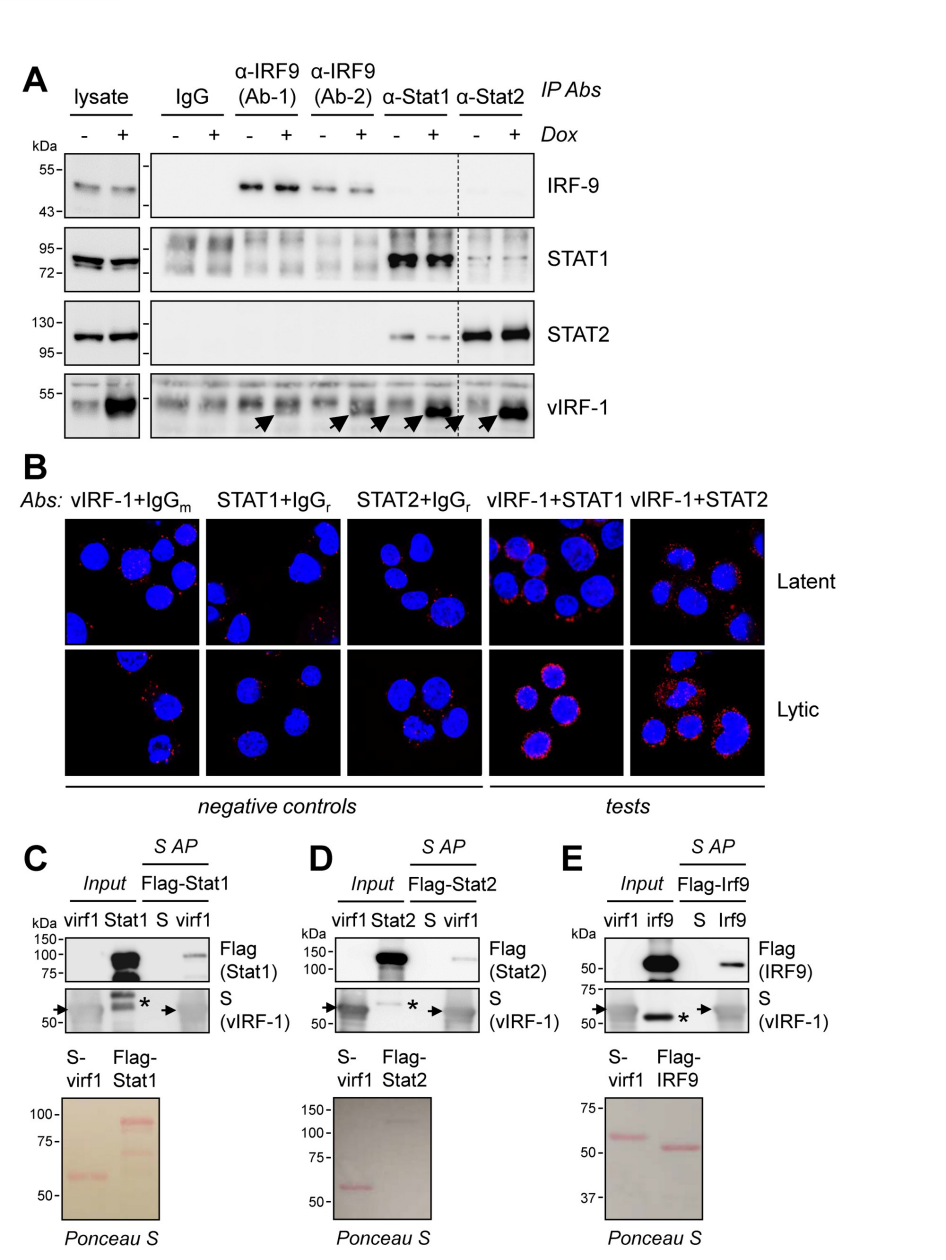
vIRF-1 interactions with ISGF3 proteins. (A) TRExBCBL1-RTA (iBCBL-1) cells, doxycycline (Dox)-inducible for viral immediate-early RTA expression [35], were either left untreated (latently infected) or treated with Dox (1 μg/ml) for 1 day to induce lytic replication. Cells were harvested and lysed for the preparation of whole-cell extracts and these were used for immunoprecipitation (IP) of IRF9, STAT1, or STAT2; for IRF9, two different IP antibodies (Ab-1, Ab-2) were used. Non-immune mouse IgG was used as a negative control. Immunoprecipitates and cell lysates were immunoblotted for detection of IRF9, STAT1, STAT2, and vIRF-1. The immunoprecipitated vIRF-1 bands, running close to unblocked Ig heavy chain (above), are indicated by arrowheads. The dotted line indicates deletion of lanes, from a single blot. (B) Colocalization of vIRF-1 with STAT1 and STAT2 was investigated by proximity ligation assay (PLA). Rabbit antiserum to vIRF-1 was paired with either rat (STAT1) or mouse (STAT2) antibody, and appropriate detection antibodies were used for PLA (see Materials and Methods). Negative controls comprised non-specific mouse IgG (IgG_m_) or rabbit IgG (IgG_r_) coupled with vIRF-1 or STAT antibodies, respectively. Cells were visualized by confocal microscopy to detect fluorescent dots, indicative of the colocalizations of complementary species-specific antibodies and therefore of the corresponding vIRF-1 and STAT targets. Untreated (latent) or Dox-induced (lytic) iBCBL-1 cells were analyzed; lytic cultures were harvested 1 day after induction. (C-E) *In vitro* coprecipitation assays using bacterially-expressed and purified STAT1, STAT2, and IRF9 (Flag-tagged) along with S-tagged and affinity-precipitated vIRF-1. Coprecipitated target proteins were detected by Flag immunoblotting, and vIRF-1-S affinity-precipitation (AP) was verified by S-peptide antibody probing (arrowheads indicate vIRF-1-S; asterisks indicate remnant Flag signal from STAT1/2 degradation products or full-length IRF9 on S-blots, following sequential probing). The input proteins used in each assay were quality-checked on Ponceau-S-stained gels (bottom panels).

Data from vIRF-1 affinity precipitation and mass spectrometry studies identified STAT3 as a potential interaction partner of vIRF-1, and this interaction was subsequently confirmed (Z. Yang, Q. Xiang, and J. Nicholas, manuscript in preparation). This prompted us to test for interactions of vIRF-1 with other members of the STAT family, specifically STAT1 and STAT2, as mediators of innate immune signaling and therefore of central importance to regulation of viral infection and replication. In transfection-based coprecipitation experiments using epitope/affinity-tagged vIRF-1, STAT1 and STAT2, we identified vIRF-1 interactions with both STATs, and similar assays identified vIRF-1 interaction also with ISGF3 co-component IRF9 (S1 Fig). Further investigations of vIRF-1 interactions with ISGF3 proteins were carried out in the context of PEL cells, naturally infected with HHV-8 and expressing vIRF-1 at low levels in latency and more abundantly in lytically reactivated cells [2,34]. Antibodies to STAT1, STAT2 and IRF9 were used for immunoprecipitation of the target cellular proteins from lysates of uninduced (latent) and lytically-reactivated TRExBCBL1-RTA cells (henceforth referred to as iBCBL-1 cells), inducible by doxycycline (Dox) treatment for immediate-early RTA-protein expression and lytic cycle reactivation [35]. Coprecipitated vIRF-1 was detected clearly in the STAT1, STAT2 and IRF9 immunoprecipitates from lysates of lytically infected cells, expressing high levels of vIRF-1, and was also detected in STAT1 and STAT2 immunoprecipitates from latently infected cells (Fig 1A). For IRF9, vIRF-1 coprecipitation from lytic-cell extracts was evident using each of two different antibodies to the target protein. The immunoprecipitation data demonstrate the interactions of naturally-expressed vIRF-1 with endogenous STAT1, STAT2 and IRF9.

For the vIRF-1-STAT interactions, we undertook proximity ligation assays to visualize colocalization of the endogenously-expressed proteins in intact iBCBL-1 cells. We found very little background signal using the vIRF-1, STAT1 or STAT2 antibodies with pre-immune mouse or rabbit IgG, but vIRF-1 antiserum (rabbit) paired with either STAT1 or STAT2 antibody (rat and mouse, respectively) led to increased abundance of fluorescent dots (Fig 1B). This was especially evident in lytically infected (Dox-treated) cells, in which vIRF-1 is induced and expressed at higher levels than in latently infected cells. Notably, both vIRF-1/STAT1 and vIRF-1/STAT2 fluorescence was detected mainly outside the nucleus, with vIRF-1/STAT1 colocalization occurring largely in perinuclear regions; vIRF-1/STAT2 dots were distributed more broadly in the cytoplasm. These data provide further evidence of vIRF-1 interactions with both STAT1 and STAT2 and identify mainly extra-nuclear localizations, and independence, of these interactions.

To test if vIRF-1 can interact with STAT1, STAT2 and IRF9 directly, coprecipitation analyses were carried out using bacterially-expressed and purified S-tagged (vIRF-1) or Flag-tagged proteins in S-protein-based affinity precipitations. These *in vitro* experiments identified coprecipitation of each of the recombinant cellular proteins with vIRF-1 (Fig 1C–1E). No precipitated STAT1, STAT2 or IRF9 was evident in S-peptide-containing control samples, demonstrating lack of detectable non-specific binding of the proteins to the affinity beads and therefore direct, bimolecular interactions between vIRF-1 and each of the ISGF3 proteins.

### Influence of vIRF-1 on IFNβ signaling in HHV-8-infected cells

**Fig 2:**
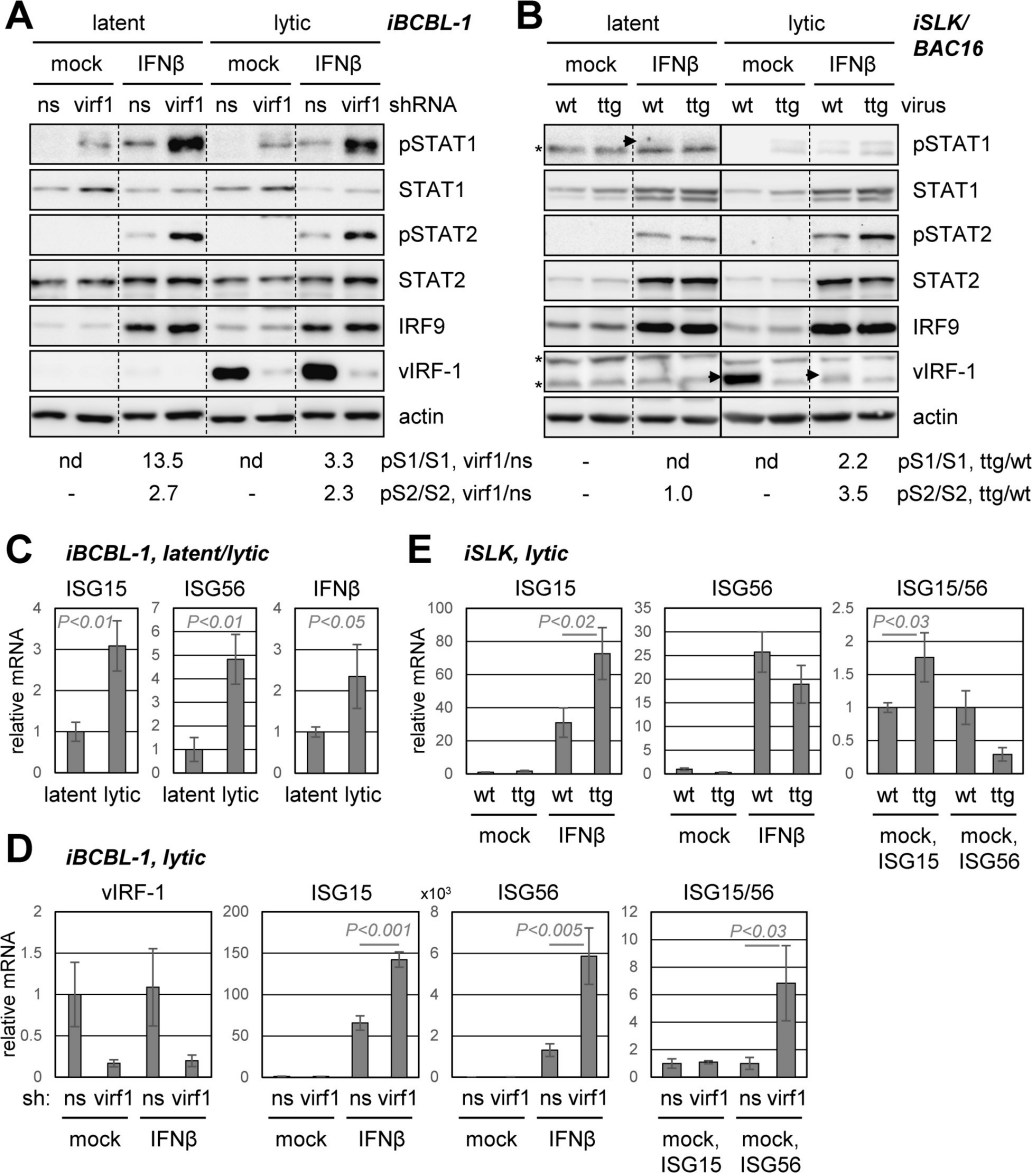
vIRF-1 regulation of STAT1, STAT2 and IFNβ signaling in HHV-8-infected cells. (A) Analysis of phosphorylated (active) STAT1 and STAT2 (pSTAT1, pSTAT2) and total STAT1 and STAT2 in latent (-Dox) and lytic (+Dox, 2 days) iBCBL-1 PEL cells, transduced with lentivirus vector expressing either non-silencing control (ns) or vIRF-1 mRNA-specific (virf1) shRNA. Cultures remained untreated (mock) or were treated with IFNβ (50 ng/ml). Derived cell extracts were immunoblotted for assessments of pSTAT1 and pSTAT2 levels, in addition to validation of vIRF-1 induction (+Dox) and depletion and monitoring of IRF9 expression. Quantified pSTAT1 (pS1) and pSTAT2 (pS2) levels for IFNβ-treated samples, normalized to total STAT1 (S1) and STAT2 (S2), respectively, and expressed as ratios of levels in vIRF-1 versus control shRNA-transduced cells (virf1/ns), are shown below the blots. Dotted lines indicate rearrangement (for consistency) and deletion of lanes. (B) Analysis of STAT1 and STAT2 regulation as a function of vIRF-1 in BAC16 HHV-8-infected iSLK cells. Cultures infected with wild-type (wt) or vIRF-1-knockout (ttg) [9] virus were treated with Dox and sodium butyrate (NaB) to induce lytic replication or left untreated (latent infection), with or without IFNβ treatment. Cells were harvested 3 days after treatment, and cell lysates were analyzed as above. Levels of pSTAT1 and pSTAT2, normalized to total STAT1 and STAT2, in ttg-virus-infected cells relative to wt-virus-infected cells are indicated below the blots. Arrowheads indicate positions of pSTAT1 and vIRF-1 bands; asterisks indicates non-specific bands. For A and B: nd, not determined; -, undetectable pSTAT. (C) Detection of lytic cycle-regulated ISG15 and ISG56 in iBCBL-1 cells. Expression of representative ISGs, ISG15 and ISG56, and also IFNβ, were assessed in latent and (1-day) lytic cells by RT-qPCR analysis of the respective transcripts. Data are from biological triplicates; error bars represent standard deviations from the means, and student t-test *P* values (two-tailed) are shown. (D) ISG15 and ISG56 transcript levels were assessed in lytically-infected iBCBL-1 cells 2 days post-Dox treatment, with or without vIRF-1 depletion, in the presence (IFNβ) or absence (mock) of IFNβ treatment (50 ng/ml). RT-qPCR for vIRF-1 mRNA confirmed vIRF-1 depletion in vIRF-1 (virf1) shRNA-expressing relative to non-silencing (ns) shRNA-expressing cells (left). (E) Equivalent analysis of ISG15 and ISG56 expression in iSLK cells infected with wild-type (wt) or vIRF-1-knockout (ttg) BAC16 virus and lytically reactivated with Dox/NaB treatment for 3 days.

In view of the interactions of vIRF-1 with components of the IFN-I-induced ISGF3 complex and the potential inhibition of these proteins and/or co-complexing by vIRF-1, we investigated the effect of vIRF-1 on IFNβ signal transduction. This was done initially in PEL cells by measuring the levels of Y_701_- and Y_690_-phosphorylated (active) STAT1 and STAT2 (pSTAT1 and pSTAT2) in response to vIRF-1 depletion, effected by lentivirally-delivered pre-validated vIRF-1-specific shRNA [9]. vIRF-1-depleted and control [non-silencing (NS) shRNA-expressing] cultures of iBCBL-1 cells were either left untreated (latent) or treated with Dox (lytic) for 2 days; additionally, one set of cultures (-/+ Dox) was treated with IFNβ, to enable identification of vIRF-1 effects on IFN-I signaling independent of any contributing activity via IFN-I suppression. Immunoblotting of cell lysates revealed that pSTAT1 levels were increased substantially by vIRF-1 depletion in both latent and lytic cultures, with or without IFNβ treatment (Fig 2A). It is known that vIRF-1 is expressed in both states in PEL cells [2,34], albeit that this was not clearly evident from the vIRF-1-blot exposure used. Although pSTAT2 was undetectable without IFNβ treatment, vIRF-1 depletion increased pSTAT2 levels in IFNβ-treated latent and lytic cultures, demonstrating that this component of the IFNβ signaling pathway is also suppressed by virus-expressed vIRF-1 in the context of PEL cells. Thus, levels of active STAT1 and STAT2 are suppressed by vIRF-1 in both phases of HHV-8 infection in this cell type, and IFNβ signaling, specifically, is inhibited by vIRF-1 in this context.

We next investigated pSTAT1 and pSTAT2 regulation by vIRF-1 in HHV-8-infected iSLK cells [36], taking advantage of a previously generated vIRF-1 knockout virus, BAC16.vIRF-1.TTG, in which the initiator ATG codon was changed to TTG [9]. Parallel iSLK cultures were infected with equivalent infectious doses of wild-type (wt) and vIRF-1-ablated (ttg) viruses, and the respective cultures were selected for latently infected cells using hygromycin treatment. These were then treated with Dox and sodium butyrate (NaB) to induce lytic replication, or left untreated (latent); one of a replicate set of cultures was treated with IFNβ. Cells were harvested to generate extracts for immunoblotting at initiation of the experiment (-Dox, latent) and at 3 days after lytic induction. Levels of pSTAT1 were unaffected by vIRF-1 ablation in latently infected cells, as expected (vIRF-1 is not expressed during latency in these cells), but were enhanced absent vIRF-1 after lytic reactivation. As in PEL cells, pSTAT2 was undetectable without IFNβ treatment, but its activation by IFNβ and suppression by vIRF-1 in this context was evident (Fig 2B). Interestingly, compared with the effect of IFNβ in lytically-infected cells, IFNβ induced much weaker (barely detectable) levels of pSTAT1 in uninduced cells, for reasons unknown. Overall, our data demonstrate the ability of virus-expressed vIRF-1 to suppress both pSTAT1 and pSTAT2 during HHV-8 lytic replication in iSLK and PEL cells and also in latently infected PEL cells, including under treatment with IFNβ, providing evidence of IFN-I-signaling suppression by vIRF-1 in infected cells.

Regulation of IFNβ signaling was further investigated in iBCBL-1 and iSLK cells by monitoring ISGF3-responsive interferon-stimulated gene (ISG) expression. Initial RT-qPCR analysis of transcripts of two representative ISGs, ISG15 and ISG56, in latently and lytically infected iBCBL-1 cells identified induction of both, along with IFNβ mRNA, in response to lytic induction (Fig 2C). These ISGs were then quantified in lytically-reactivated iBCBL-1 cells in response to vIRF-1 depletion. Levels of both ISG mRNAs were enhanced in vIRF-1-depleted relative to control (NS shRNA-expressing) iBCBL-1 cells in IFNβ-treated cultures, and ISG56 transcripts were induced by vIRF-1 depletion also in untreated (mock) cells (Fig 2D). These data are consistent with identified pSTAT1- and pSTAT2-suppressive activity of vIRF-1 (Fig 2A). Similar analyses were carried out in iSLK cells infected with either wild-type (wt) or vIRF-1-knockout (ttg) BAC16 virus. ISG15 mRNA expression was increased in response to vIRF-1 ablation, but, in these cells, ISG56 transcript levels were unaltered or reduced (+/- IFNβ treatment) (Fig 2E). The “negative” results for ISG56 may be due to cell-type specificity of ISG expression and regulation. Overall, the RT-qPCR data provide supporting evidence of vIRF-1 suppression of IFN-I signaling, affecting gene expression downstream of pSTAT1 and pSTAT2 regulation.

### Suppression of interferon signaling by vIRF-1 in transfected cells

**Fig 3:**
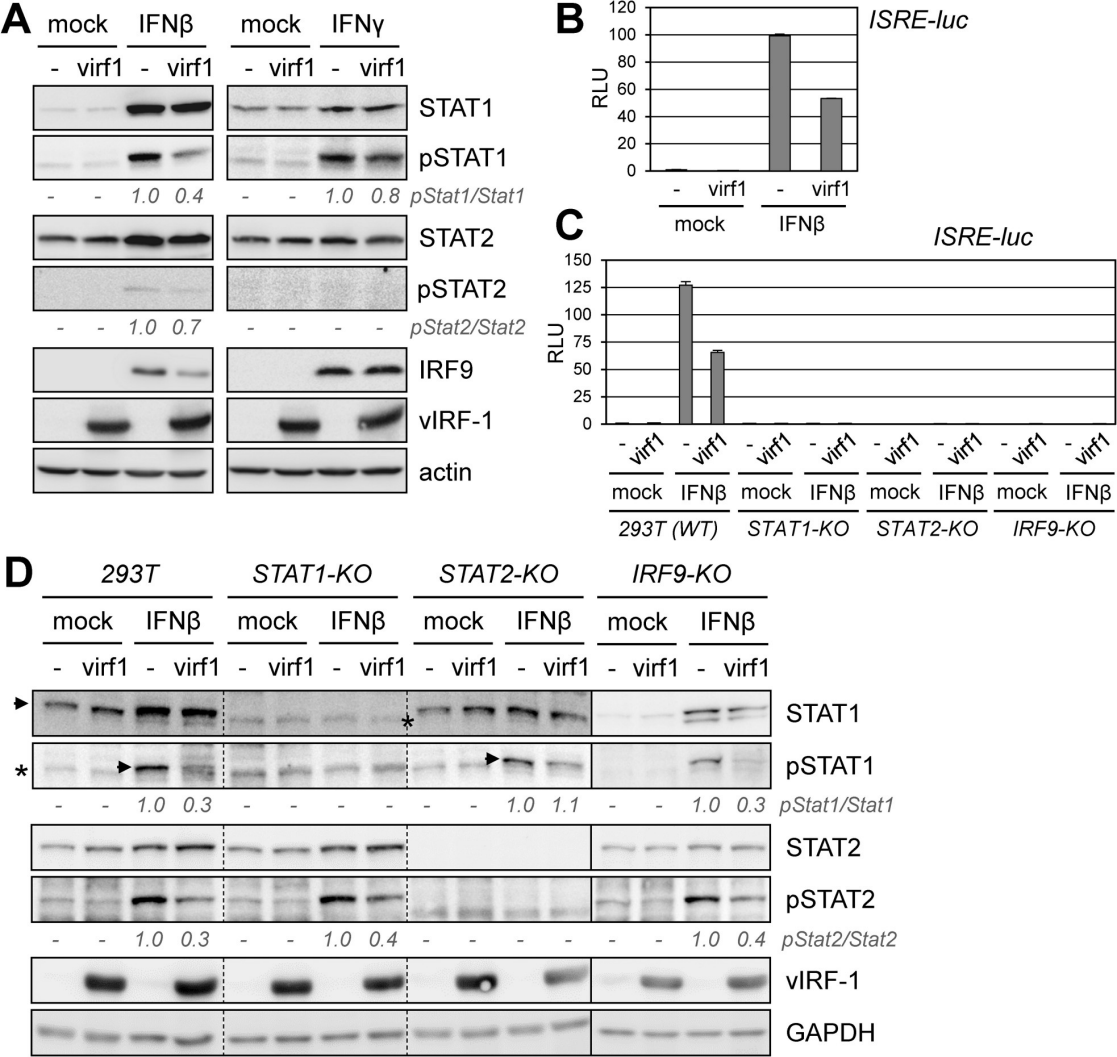
Regulation of IFNβ signaling by vIRF-1 in transfected cells. (A) 293T cells transfected with empty vector (-) or vIRF-1 expression plasmid (virf1) were left untreated or treated with IFNβ or IFNγ (50 ng/ml of each) for 24 h. Cells were then harvested and lysates analyzed by immunoblotting for relative levels of pSTAT1 and pSTAT2, total STAT1 and STAT2, and IRF9. As applicable (detected), pSTAT1/STAT1 and pSTAT2/STAT2 ratios (“empty vector” values set at 1) are shown below the respective blots for IFNβ- and IFNγ-treated cells. (B) vIRF-1 suppression of IFNβ-induced signaling, as measured by ISRE-luciferase (luc) reporter assay, was detected in 293T cells cotransfected with the reporter and empty vector (-) or expression plasmid for vIRF-1 (virf1), left untreated or treated with IFNβ (50 ng/ml) for 24 h. Relative luciferase assay-derived luminescence values (RLU, means from duplicate transfectants) are shown along with standard deviations from the means. Student’s t-test *P* value (unpaired, two-tailed) for vIRF-1 suppression of IFNβ signaling (vIRF-1-vector relative to empty-vector RLU) is 0.0005. (C) STAT1, STAT2, and IRF9 knockout (KO) 293T cell lines were generated by Cas9/gRNA transduction, blasticidin selection, and clonal isolation of cells (see Materials and Methods). Target-gene ablation in each of the cell lines was tested functionally by ISRE-luciferase reporter assay following IFNβ stimulation (chart; N = 2). Mock, untreated cultures; virf1, vIRF-1 vector-transfected cells; -, empty vector-transfected cells. (D) vIRF-1 suppression of IFNβ-activated STAT1 and STAT2 (pSTAT1, pSTAT2) as a function of STAT1, STAT2, and IRF9 knockout. Cultures were transfected with empty vector (-) or vIRF-1 expression plasmid (virf1) and, after 6 h, treated with IFNβ (50 μg/ml) for 24 h. Cell lysates were analyzed by immunoblotting; numbers below the pSTAT1 and pSTAT2 blots show quantified pSTAT1 and pSTAT2 levels normalized to total STAT1 and STAT2, respectively, in the absence (value set at 1) and presence of vIRF-1. Arrows, STAT1/pSTAT1; asterisks, non-specific bands; dotted lines, lane deletions from single blot; solid line, different membrane.

As a tractable system in which to assess mechanisms of vIRF-1 regulation of STAT1, STAT2 and ISGF3 complexing and signaling, we used 293T cells for transfection-based studies. First, we assessed pSTAT1 and pSTAT2 levels, in addition to ISGF3-induced IRF9, as a function of vIRF-1 expression and treatment with IFNβ. Immunoblotting for phosphorylated and total STAT1 and STAT2 revealed the suppression of IFNβ-activated STATs 1 and 2 by vIRF-1, but a relatively modest reduction of IFNγ-induced pSTAT1 in the presence of vIRF-1 (Fig 3A). Consistent with pSTAT1 and pSTAT2 suppression by vIRF-1, the viral protein effected reduced levels of IFNβ-induced IRF9. Reflective of the immunoblot data, and in agreement with previously published findings from reporter-based assays [11,33], vIRF-1 inhibited ISGF3-luciferase reporter-detected IFNβ signaling in transfected 293T cells (Fig 3B). Consistent with the results from infected cells (Fig 2), these data identify vIRF-1 as an antagonist of IFNβ via suppression of pSTAT1 and pSTAT2, potentially mediated by interactions of vIRF-1 with one of more of the ISGF3 components.

We next assessed the requirements for each of the ISGF3 proteins, STAT1, STAT2 and IRF9, for vIRF-1-mediated suppression of IFNβ-induced levels of pSTAT1 and pSTAT2. This was done using custom-engineered 293T cell lines genetically deficient for STAT1, STAT2 or IRF9 (see Materials and Methods). The engineered cell lines, found by immunoblotting to be negative for expression of the respective proteins, were also tested by ISRE-luciferase reporter assay for negation of IFNβ signaling (Fig 3C); of note, transfectants expressing vIRF-1 also were negative for IFNβ signaling, demonstrating lack of functional complementation of any of the ISGF3 components by the viral protein. In STAT1-knockout (KO) cells, IFNβ-activated pSTAT2 was suppressed by vIRF-1, as in the parental wild-type cells (Fig 3D). In the context of STAT2-KO cells, IFNβ treatment led to increased pSTAT1 (absent increased total STAT1, observed in wild-type cells), but there was a loss of vIRF-1-mediated pSTAT1 suppression (which was confirmed in other experiments). In IRF9-KO cells, pSTAT1 and pSTAT2 levels were elevated in response to IFNβ treatment, and vIRF-1 was able to suppress each. Taken together, these data reveal that STAT2 plays a role in the suppression of pSTAT1 by vIRF-1, that IRF9 has little influence on this process, and that pSTAT2 suppression by vIRF-1 occurs independently of STAT1 and IRF9; the data suggest that STAT1-STAT2 interaction, rather than ISGF3 complexing, *per se*, is of primary importance for pSTAT1 regulation by vIRF-1 and that pSTAT2 suppression occurs by a distinct mechanism.

### Activation versus decay of pSTAT1 and pSTAT2 in response to vIRF-1

**Fig 4:**
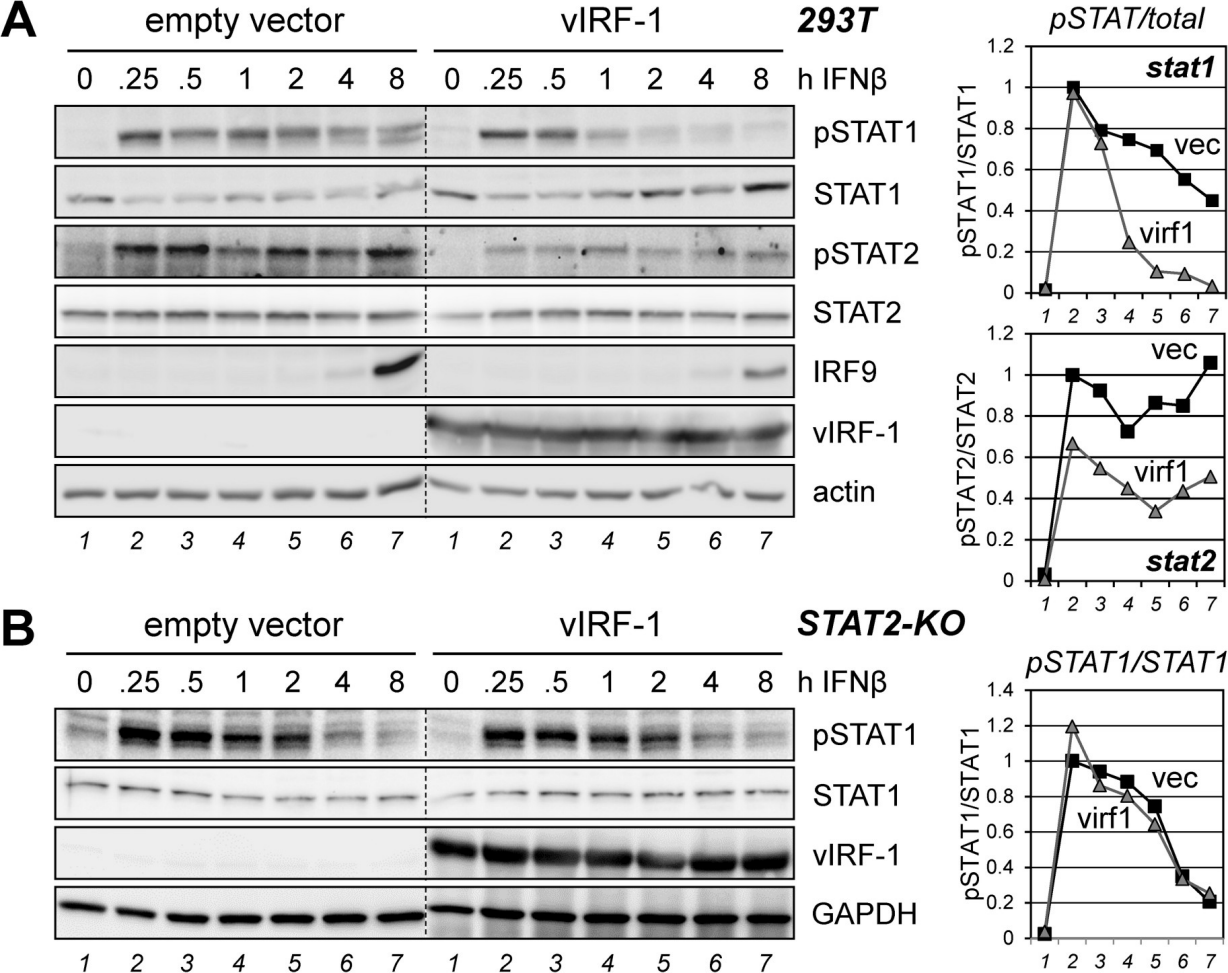
Effects of vIRF-1 on the amplitudes and kinetics of IFNβ-activated STAT1/2. (A) 293T cells transfected with empty vector (control) or vIRF-1 expression plasmid were left untreated (0 h) or treated with IFNβ (50 ng/ml) for different times (0.25 to 8 h). Cells were harvested, lysed, and analyzed by immunoblotting for detection of phosphorylated and total STAT1 and STAT2, IRF9, and also vIRF-1, to check expression in the transfectants. Relative levels of pSTAT1 and pSTAT2, normalized to STAT1 and STAT2, respectively, and with empty vector (vec) values at 0.25 h set at 1, are shown in the adjacent charts. (B) An analogous experiment was performed in STAT2-knockout (KO) 293T cells. The immunoblot-derived pSTAT1 levels, normalized to STAT1 and expressed relative to vector/0.25 h pSTAT1 (set at 1), are plotted in the chart (right). For both panels, dotted lines indicate lane deletion or rearrangement of blot sections for consistency of presentation.

The preceding analyses indicated that STAT1 complexing with STAT2, alone or in association with IRF9, may be important for suppression of pSTAT1 by vIRF-1, but that pSTAT2 is regulated differently. To ty to gain further insight into these processes, we assessed the effect of vIRF-1 on the amplitudes and kinetics of STAT1 and STAT2 phosphorylation (activation) and dephosphorylation after IFNβ treatment. We treated 293T cultures, transfected with empty vector or vIRF-1 expression plasmid, with IFNβ for 0.25 to 8 h, and harvested the respective cultures for immunoblotting of cell lysates. For STAT1, there was little effect of vIRF-1 on initial activation (pSTAT1 levels at 0.25 and 0.5 h post-IFNβ), but vIRF-1 effected a markedly more rapid “decay” of pSTAT1 (Fig 4A). Thus, while pSTAT1 levels were sustained at 8 h post-treatment at approximately 60% of the initially induced level (at 0.25 h) in the empty-vector transfectants, the level of pSTAT1 at the 8 h timepoint was barely detectable in the vIRF-1 expressing cells and reduced to around 25% of maximum (at 0.25 h) by 1 h. In contrast, initial IFNβ-induced phosphorylation of STAT2 was decreased by vIRF-1 (to ~67% of empty-vector control), but induced pSTAT2 levels were largely sustained over the 8 h experimental period. Consistent with the suppression of pSTAT1 at later times and overall suppression of pSTAT2, IFNβ-induced IRF9 expression [37], detectable at the last timepoint, was reduced in the presence of vIRF-1 (~36% of actin-normalized level in empty-vector-transfected cells). In an analogous experiment using 293T STAT2-KO cells, IFNβ induced similar levels of pSTAT1 in the absence or presence of vIRF-1, as in wild-type cells, but vIRF-1 had no detectable effect on pSTAT1 decay in these cells (Fig 4B). These data demonstrate the involvement of STAT2 in vIRF-1 promotion of pSTAT1 dephosphorylation (or conceivably degradation) following IFNβ stimulation, an activity of vIRF-1 likely dependent on co-activation of and complexing between pSTAT1 and pSTAT2, with or without IRF9 co-association.

### Influence of vIRF-1 on ISGF3-protein interactions

**Fig 5:**
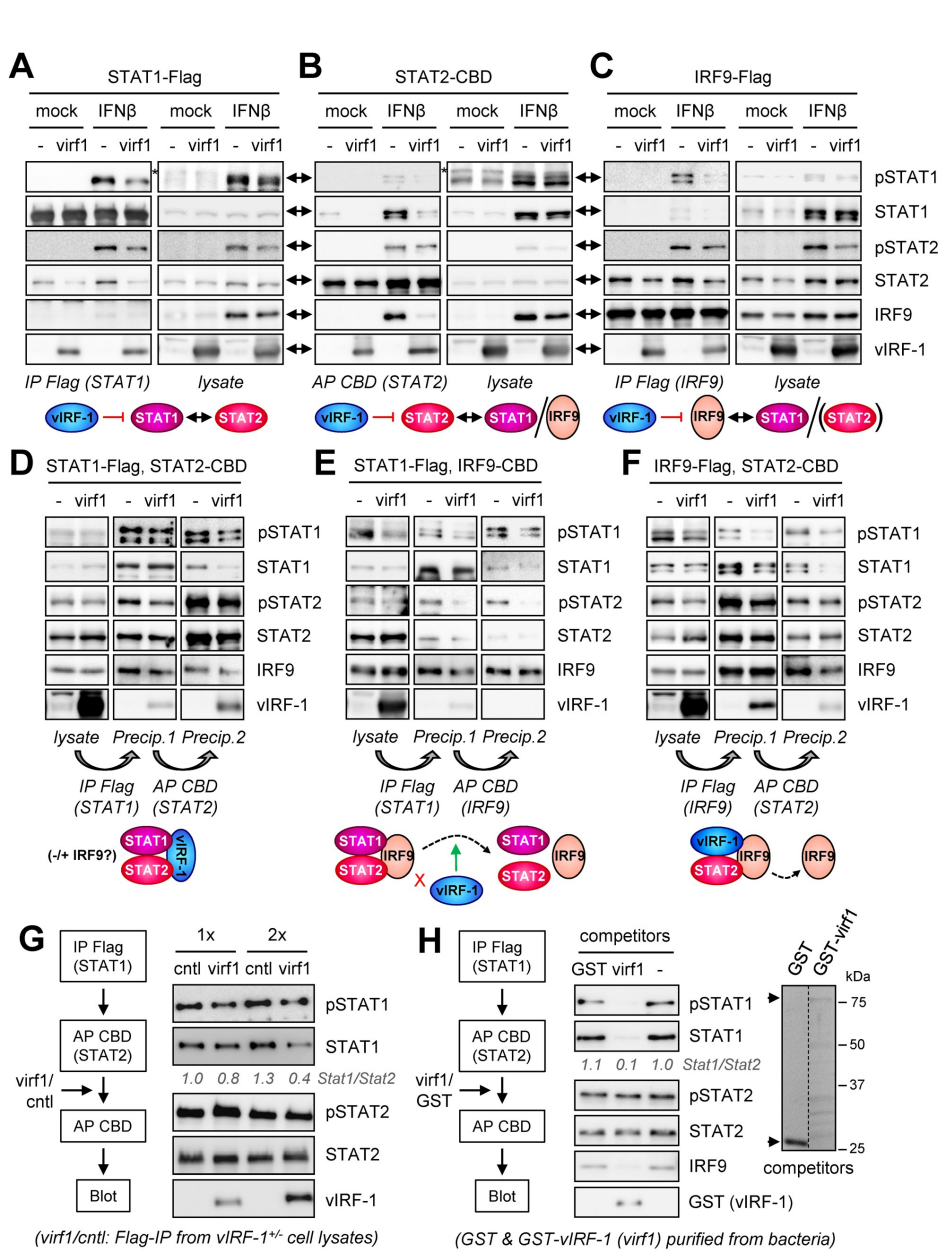
Effects of vIRF-1 on complexing of ISGF3 components. (A-C) Flag-tagged STAT1 (A) or IRF9 (C) or CBD-tagged STAT2 (B) were expressed in 293T cells transfected with the respective expression plasmids and either vIRF-1 (virf1) or empty (-) expression vector; replicates were either left untreated (mock) or treated with IFNβ (10 ng/ml) for 24 h. Flag/CBD-tagged proteins were then immuno/affinity-precipitated from cell lysates, and coprecipitated ISGF3 proteins were detected by immunoblotting. Diagrams below the panels illustrate the main findings from each experiment. (D-E) Serial coprecipitations of STAT1 and STAT2 (D), STAT1 and IRF9 (E), and IRF9 and STAT2 (F), respectively Flag- and CBD-tagged, from lysates of transfected 293T cells expressing vIRF-1 (virf1) or cotransfected with empty vector (-). All transfectants were treated with IFNβ for 24 h prior to harvesting. First (Flag IP) and second (CBD AP) precipitates (Precip. 1, Precip. 2) were analyzed by immunoblotting for the tagged “bait” proteins, the third (endogenous) ISGF3 protein (including phosphorylated and total STAT1 and STAT2), and vIRF-1; lysates were immunoblotted for expression of input proteins. Illustrated below each panel of blots are the main findings. (G) Disruption by vIRF-1 of STAT1-STAT2 complexes isolated by Flag-IP (STAT1) and CBD-AP (STAT2) from IFNβ-treated transfected 293T cells. Immunoprecipitated material from vIRF-1-Flag (virf1) or empty control (cntl) vector-transfected cells was applied in two concentrations (1x, 2x) to dual-precipitation-derived STAT1/STAT2 complexes, and then mixtures were subjected to re-precipitation with chitin beads (binding STAT2-CBD). STAT1 and vIRF-1 associated with re-precipitated STAT2-CBD were identified by immunoblotting. Relative levels of co-precipitated STAT1, normalized to affinity-sedimented STAT2, are shown below the STAT1 blot (cntl/1x value set at 1). (H) An equivalent experiment was carried out using GST-fused recombinant vIRF-1 (virf1) or GST (negative control) to challenge STAT1 interaction with STAT2 in STAT1/STAT2 hetero-complexes isolated by IP/AP dual precipitations from IFNβ-treated 293T cells. Endogenous IRF9 interaction with STAT1/2 and competition by vIRF-1 were also monitored. Relative levels and integrities of the recombinant proteins are shown in the Coomassie-stained gel (right); arrowheads indicate the positions of the full-length proteins.

The dependence of vIRF-1-mediated pSTAT1 suppression on IFNβ-co-activated STAT2 (Figs 3D and 4B) and involving accelerated diminution of pSTAT1 post-activation (Fig 4A) indicated that vIRF-1, via its interactions with STAT2 and/or STAT1, might inhibit pSTAT1 complexing with pSTAT2, as part of ISGF3, or promote dissociation of pSTAT1 from these complexes, thereby accelerating dephosphorylation of pSTAT1. It has been reported, for example, that mutation-effected stabilization of active pY-SH2-associated (parallel) pSTAT1 dimers renders pSTAT1 less susceptible to dephosphorylation, while parallel-dimer disruption by overexpression of the STAT1 N-terminal domain effects the reverse [38]. Thus, pSTAT1 in active pSTAT1:pSTAT2 dimers occurring in ISGF3 would be expected to be more resistant to dephosphorylation than uncomplexed pSTAT1. To investigate the effect of vIRF-1 on STAT1-STAT2 interaction, we carried out transfection-based coprecipitation assays, using Flag- or chitin-binding domain (CBD)-tagged STAT1 or STAT2, which were immuno/affinity-precipitated from cell lysates; cells were stimulated with IFNβ or left untreated. In STAT1 precipitations, vIRF-1-reduced levels of STAT2 (and IRF9) coprecipitated from lysates of IFNβ-stimulated cells correlated with suppressed pSTAT1 and pSTAT2 levels in cell lysates (Fig 5A). However, in STAT2 precipitations, vIRF-1 effected a more pronounced decrease in STAT2-associated pSTAT1/STAT1, and also IRF9, than of pSTAT1 and pSTAT2 (Fig 5B), indicating direct interference with STAT1-STAT2 and STAT2-IRF9 interactions, independent of pSTAT1/2 regulation. Both STAT1 and STAT2 precipitations detected interaction of unphosphorylated STAT1 and STAT2 (but not STAT1/2-IRF9 associations) in the absence of IFNβ stimulation and this was inhibited by vIRF-1, providing evidence of direct suppression of STAT1-STAT2 binding by vIRF-1. The association of vIRF-1 with both STATs independently of IFNβ stimulation is consistent with such suppression and may contribute to inhibition of pSTAT1-pSTAT2 association following IFNβ treatment. Using IRF9-Flag as bait in another experiment, we detected vIRF-1 inhibition of pSTAT1/STAT1-IRF9 interaction beyond the level of pSTAT1 suppression (in lysate), but no apparent diminution of pSTAT2 binding relative to reduced pSTAT2 (Fig 5C). However, binding of total-STAT2 to IRF9 was detectably diminished by vIRF-1 in both IFNβ-treated and unstimulated cells; it is known that unphosphorylated STAT2 can interact with IRF9 [39,40]. As for STAT1 and STAT2, vIRF-1 associated with IRF9 in unstimulated cells, revealing further potential for vIRF-1 interference with ISGF3 complex formation. Combined, the precipitation data identify inhibitory effects of vIRF-1 on STAT1-STAT2, IRF9-STAT1 (in ISGF3), and IRF9-STAT2 interactions that are independent of pSTAT1/2 suppression.

To investigate vIRF-1 effects on ISGF3-protein complexing, we carried out a series of tandem precipitation assays, using differently-tagged ISGF3 protein pairs from IFNβ-treated transfected cells expressing or lacking vIRF-1. In STAT1-STAT2 dual precipitations, vIRF-1-associated reductions in IRF9 and pSTAT2/STAT2 levels in the first and second precipitates correlated with decreased overall pSTAT1/2 (relative to total STAT1/2) detected in cell lysates (Fig 5D). Detection of vIRF-1 in final STAT1/STAT2 precipitates demonstrated the ability of vIRF-1 to associate with STAT1-STAT2 complexes. In STAT1-IRF9 serial precipitations, STAT2 and IRF9 interactions with STAT1 (first precipitation) were reduced, along with pSTAT1/2, by vIRF-1, reflective of reduced ISGF3 complexing (Fig 5E). However, in secondary, IRF9 precipitates from STAT1-selected material, and therefore representing ISGF3, pSTAT1/STAT1 and pSTAT2/STAT2 levels relative to precipitated IRF9 were reduced substantially in the presence of vIRF-1, suggesting destabilization of ISGF3 complexes by vIRF-1. The absence of detectable vIRF-1 in final precipitates indicates that vIRF-1 cannot co-complex with STAT1 and IRF9, and by extension ISGF3, STAT2 being required for STAT1-IRF9 association [39]. In analyses of IRF9-STAT2 complexes, vIRF-1 effected diminished IRF9 levels, relative to precipitated STAT2, in the second precipitates (Fig 5F); these data reveal destabilization of IRF9-STAT2 interaction by vIRF-1 between the first (IRF9) and second (STAT2) precipitations. Detection of vIRF-1 in the final precipitates demonstrated complexing between vIRF-1, IRF9 and STAT2. Taken together, the serial precipitation data show that vIRF-1 can co-associate with STAT1-STAT2 and IRF9-STAT2, but not IRF9-STAT1 (ISGF3), complexes and provide evidence that vIRF-1 can compete for and/or destabilize STAT1 and STAT2 interactions with IRF9.

To test directly if vIRF-1 can disrupt ISGF3 complexes, thereby enabling dephosphorylation of pSTAT1 and accounting (at least in part) for pSTAT1 suppression by vIRF-1, we assessed the effect of vIRF-1 on IFNβ-induced and isolated STAT1-STAT2 complexes. STAT1-Flag and STAT2-CBD, expressed in transfected 293T cells treated with IFNβ, were precipitated serially from derived cell extract, and then the isolated STAT1/STAT2-containing complexes were challenged with immuno-purified vIRF-1-Flag. Following incubation, the resulting complexes were further precipitated with chitin beads for isolation of STAT2-CBD prior to immunoblotting for detection of coprecipitated STAT1. The results (Fig 5G) revealed vIRF-1 competition for (activated) STAT1 association with STAT2. An equivalent experiment was carried out using bacterially-expressed and purified GST (negative control) and GST-vIRF-1 proteins as the competitors, or no challenge. GST-vIRF-1, but not GST, competed STAT1, and also endogenous IRF9, interactions with STAT2 (Fig 5H). Thus, vIRF-1 has the ability to disrupt IFNβ-induced pSTAT1-pSTAT2 interactions (in ISGF3 complexes), effecting release of pSTAT1 and thereby increasing its susceptibility to dephosphorylation. It is also possible that vIRF-1 can inhibit ISGF3 formation via interaction with STAT1, STAT2 and/or IRF9, but our data do not provide specific evidence of this.

### Janus kinases as targets of vIRF-1 interaction and function

**Fig 6:**
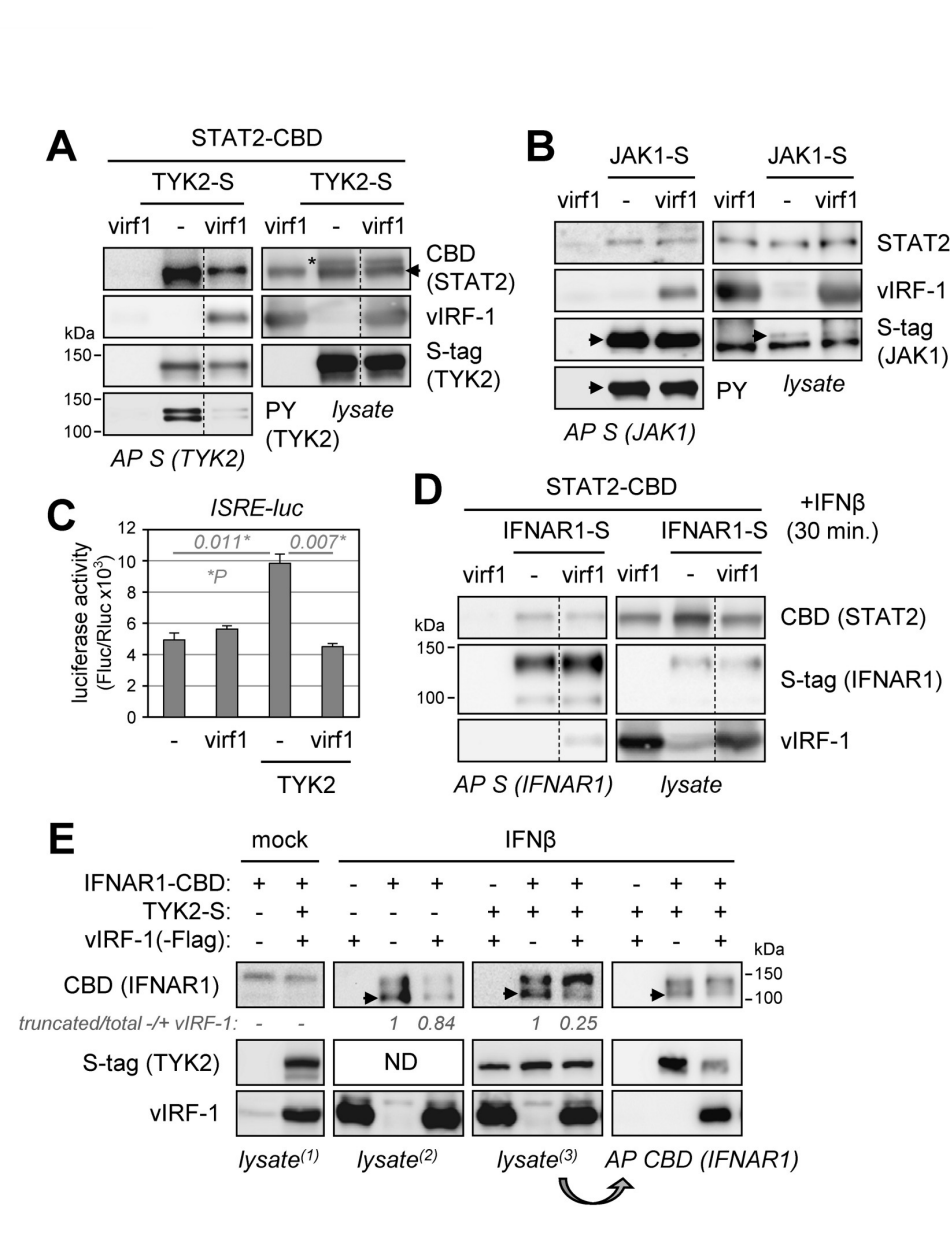
Physical and functional interactions of vIRF-1 with JAKs. (A) TYK2-S and STAT2-CBD were expressed with (virf1) or without (-) vIRF-1 in transfected 293T cells. Cell lysates and S-protein affinity-precipitates were assessed for input protein expression and sedimentation by immunoblotting with CBD (STAT2), vIRF-1, and S-tag (TYK2) antibodies. Affinity-precipitated TYK-2-S was probed with phospho-tyrosine (PY)-specific antibody to identify the active, autophosphorylated form of the kinase. Dotted lines indicate lane deletions from single membranes; the arrowhead and asterisk indicate CBD-specific (STAT2) band and remnant S-tag signal (after blot stripping), respectively. (B) An equivalent experiment was performed to assess vIRF-1 effects on JAK1 autophosphorylation and association with STAT2. Here, STAT2 antibody was used to detect endogenous protein. Arrowheads indicate JAK1-S (~130 kDa). (C) ISRE-luciferase reporter assay to assess vIRF-1 inhibition of TYK2-mediated signal transduction in 293T cells cotransfected with TYK2-expression and reporter plasmids and either vIRF-1 (virf1) or empty (-) expression vectors. Average values from duplicate samples for each condition are shown; error bars indicate standard deviations from the means. Statistical significance (P) was determined by student t-test (two-tailed, unpaired). (D) IFNAR1-S-based coprecipitation assay to test the influence of vIRF-1 (virf1), relative to empty-vector (-) transfection, on STAT2-receptor association, following IFNβ stimulation for 30 min. STAT2-CBD vector cotransfection provided expression of STAT2 above endogenous levels, to facilitate detection. (E) Effect of vIRF-1 on IFNβ receptor (IFNAR1) activation and association with TYK2. Transfectants expressing vIRF-1 or containing empty vector (-, negative control) and expressing, or lacking (-), introduced IFNAR1-CBD were left untreated (mock) or treated with IFNβ (10 ng/ml) for 24 h; TYK2-S was expressed in a subset of the transfected cultures. Cell lysates were analyzed for expression of the introduced proteins, and IFNAR1-CBD was affinity-precipitated from a subset of lysates to assess interaction of the receptor with TYK2 in response to vIRF-1. The numbers below the CBD blots show relative levels (-/+ vIRF-1) of IFNβ-induced lower IFNAR1 band (arrowheads) to total IFNAR1 (top plus bottom bands) from TYK2-overexpressing transfectants (+TYK2) and those devoid of TYK2 expression plasmid (-TYK2); values in the absence (-) of vIRF-1 are set at 1. For all precipitations (panels A, B, D and E), cultures were treated with DSP (2 mM, 30 min.) immediately prior to cell harvest, to stabilize targeted complexes.

In contrast to the promotion by vIRF-1 of pSTAT1 dephosphorylation following activation by IFNβ, vIRF-1 effected decreased activation of STAT2, with little effect on pSTAT2 decay (Fig 4A). This suggested that vIRF-1 may affect interactions associated with phosphorylation of STAT2. Therefore, we examined if vIRF-1 could alter interactions between STAT2 and activating Janus kinases TYK2 and JAK1, associated with IFN-I-receptor signaling. For examination of TYK2-STAT2 interaction, each affinity/epitope-tagged protein was expressed in transfected cells with or without vIRF-1 coexpression. Immunoblot analyses of TYK2-S co-precipitates revealed that TYK2 association with STAT2 and also TYK2 phosphorylation, resulting from its overexpression, were reduced substantially in the presence of vIRF-1 (Fig 6A). In a similar experiment employing S-tagged JAK1, JAK1 interaction with (endogenous) STAT2 and phosphorylation of JAK1 were not affected by vIRF-1 (Fig 6B). Reflective of the inhibition of (IFNβ signaling-independent) TYK2 activity and/or TYK2-STAT2 interaction by vIRF-1, assessment of TYK2-activated signaling by ISRE-luciferase reporter assay revealed suppression by vIRF-1 in untreated cells (Fig 6C). Together, these data identify inhibitory activity of vIRF-1 specifically on TYK2-mediated signal transduction.

The potential effect of vIRF-1 on IFNβ-induced STAT2 association with IFNβ receptor IFNAR1 was assessed in another coprecipitation experiment, employing affinity-captured S-peptide-tagged IFNAR1. vIRF-1 was found to associate with IFNAR1, likely via TYK2 and/or STAT2, and had no detectable effect on IFNAR1-STAT2 binding (Fig 6D), indicating that this is not a means of vIRF-1 inhibition of STAT2 activation. A related experiment was carried out to assess the potential influence of vIRF-1 on TYK2 association with IFNAR1. Here, IFNAR1-CBD was affinity precipitated from transfected-cell lysates prior to immunoblotting for detection of TYK2-S. The amount of coprecipitated TYK2 was diminished substantially in the presence of vIRF-1 (Fig 6E, right). The inhibition of IFNAR1-TYK2 association by vIRF-1, in addition to vIRF-1 inhibition of TYK2 activity (Fig 6A), indicates that vIRF-1 suppression of IFNβ signaling can occur upstream of vIRF-STAT interactions. In this experiment, we observed a faster-migrating IFNAR1-CBD band correlating specifically with IFNβ treatment, and this IFNβ-induced band, as a proportion of total IFNAR1, was reduced substantially by vIRF-1 in the presence of TYK2-S (Fig 6E, “lysate^(3)^” blot). These data are consistent with IFNβ-induced activation (phosphorylation) of IFNAR1 being inhibited via vIRF-1-mediated TYK2 inhibition and/or dissociation of TYK2 from the receptor. Of relevance to the latter mechanism is the (non-catalytic) role of TYK2 in stabilizing plasma membrane expression of IFNAR1 by inhibiting its internalization and degradation [41]; vIRF-1 expression absent TYK2 overexpression led to reduced levels of IFNAR1 (Fig 6E, “lysate^(2)^” blot). Interestingly, vIRF-1 had little influence on the ratio of full-length to truncated IFNAR1 under these conditions, contrasting with the clear effect of vIRF-1 in the presence of overexpressed TYK2; the basis of this difference remains to be determined, but demonstrates TYK2 involvement in vIRF-1 activity.

## Discussion

**Fig 7:**
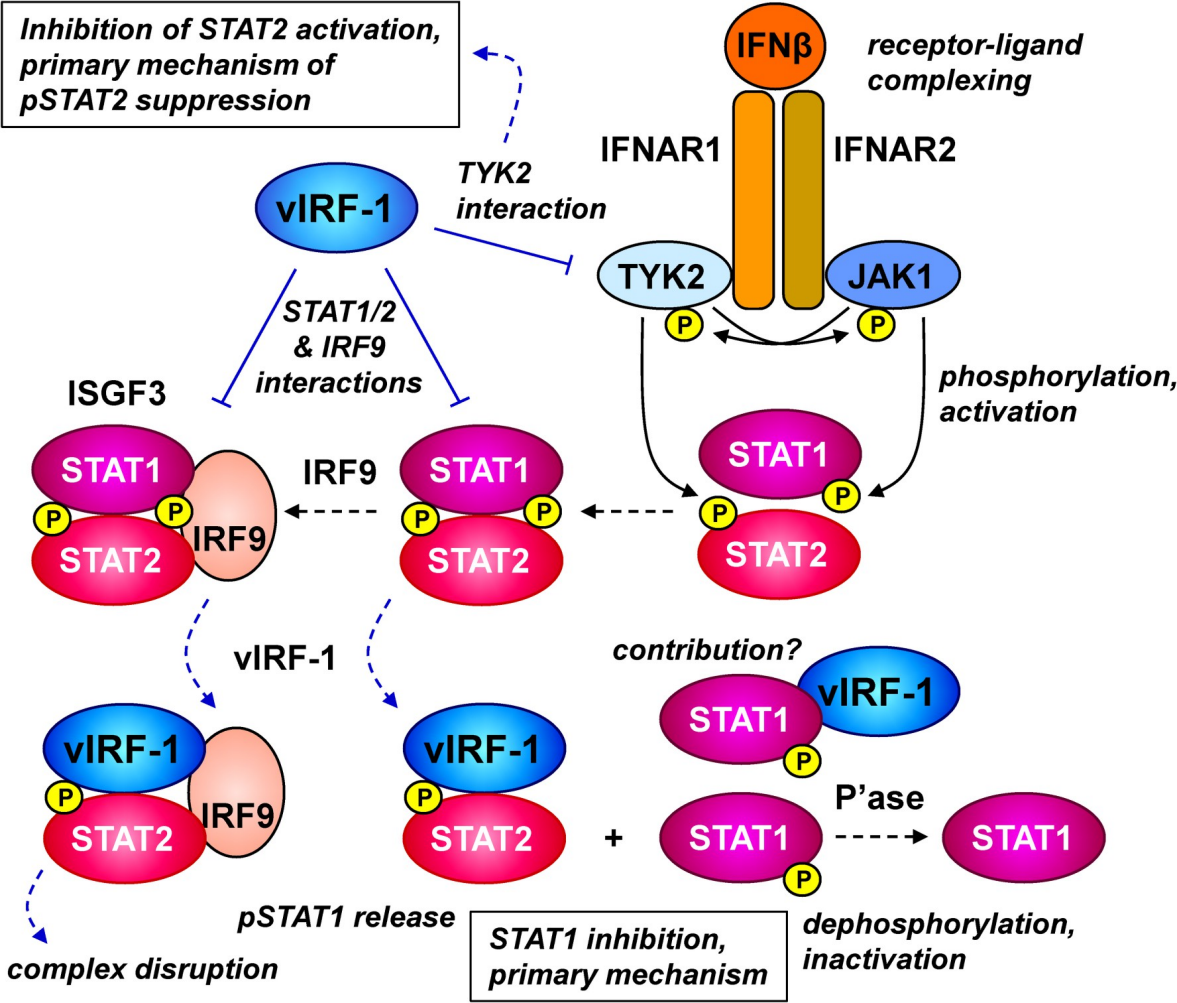
Model of vIRF-1 inhibition of IFN-β signaling based on the presented data. IFNβ binds to IFNAR1 and IFNAR2 to effect receptor activation through auto/cross-phosphorylation of receptor associated JAK kinases (JAK1 and TYK2) and receptor tyrosine residues. STAT1 and STAT2 are recruited and activated through JAK-mediated tyrosine phosphorylation, and then can heterodimerize and associate with IRF9 to form nuclear-localizing and transcriptionally active ISGF3 complexes. Data presented here show that vIRF-1 binds directly to STAT1, STAT2 and IRF9 and can also associate, directly or indirectly, with TYK2 and JAK1. vIRF-1 inhibits TYK2, but not JAK1, autophosphorylation (activation), likely contributing to pSTAT2 suppression; vIRF-1 also inhibits TYK2-STAT2 and TYK2-IFNAR1 association (not illustrated). However, suppression of pSTAT1 is mediated largely after IFNβ-induced STAT1 phosphorylation, involving increased rates of pSTAT1 decay, likely via phosphatase (P’ase)-mediated dephosphorylation (rather than pSTAT1 degradation). Suppression of pSTAT1 by vIRF-1 is dependent on STAT2, being negated by STAT2 knockout in 293T cells; this along with decreased IFNβ-stimulated pSTAT1 association with STAT2 and IRF9 in the presence of vIRF-1 indicates that vIRF-1 may suppress pSTAT1 via release of the transcription factor from ISGF3 or STAT1-STAT2 heterodimers, thereby promoting dephosphorylation of pSTAT1. Detection of intracellular complexes containing vIRF-1, pSTAT2 and IRF9 and dissociation of IFNβ-induced STAT1-STAT2 interaction by vIRF-1 *in vitro* are consistent with this model. Data from *in vitro* competition and STAT1/IRF9 and IRF9/STAT2 serial precipitation experiments provide evidence that vIRF-1 may also destabilizes IRF9-STAT2 association and dissociate ISGF3 interactions. It is possible that vIRF-1 also inhibits ISGF3 complex formation (not illustrated), via (detected) interactions with pre- and/or post-activated STAT1, STAT2 and IRF9, but the presented data do not specifically address this. In the diagram, blue lines indicate activities and consequences of vIRF-1.

As outlined in the Introduction and reviewed elsewhere [6,7], various cellular proteins have been reported to interact with vIRF-1. In addition, vIRF-1-based coprecipitation or proximity biotin labeling assays coupled with mass spectrometry have identified other candidate interactors [42,43]. The work presented here has identified new vIRF-1 interactions, with innate-immune signaling mediators STAT1 and STAT2, in addition to STAT-activating Janus kinase TYK2, and inhibitory effects on these proteins via suppression of TYK2 activity, pSTAT1 and pSTAT2 levels, and ISGF3 complex formation and stability. While it has been reported that HHV-8 vIRF-2 is able to effect the suppression of IFNα-induced ISGF3 complex formation, pSTAT1 levels, and associated IRF9 expression [44], the present study is the first report of direct interactions of any vIRF with STAT proteins, and IRF9, and the ability of vIRF-1 to disrupt IFN-I-induced STAT1/2 complexes. We have also identified distinct mechanisms by which vIRF-1 suppresses IFNβ-induced pSTAT1 and pSTAT2 levels, with the former being dependent on STAT2 and likely mediated by vIRF-1-effected release of pSTAT1 from pSTAT2-containing complexes (including ISGF3), thereby enabling pSTAT1 dephosphorylation, and the latter likely effected predominantly via inhibition of TYK2 and/or TYK2 interactions with STAT2 and IFNAR1. Thus, vIRF-1 suppression of IFNβ-induced pSTAT1 was found to be abolished in STAT2-null 293T cells (Fig 3D), vIRF-1 inhibited intracellular pSTAT1 association with STAT2 and disrupted the interaction *in vitro* (Fig 5), and TYK2 autophosphorylation and coprecipitation with STAT2 and IFNAR1 were inhibited by vIRF-1 (Fig 6A and 6E). Mechanistic differences of suppression of pSTAT1 and pSTAT2 by vIRF-1 were also apparent from the differences in vIRF-1-modulated activation and duration of IFNβ-induced pSTAT1 versus pSTAT2; the former was notably affected with respect to duration (more rapid pSTAT1 decay, likely via dephosphorylation) but not initial activation, whereas the reverse was true of pSTAT2, with overall decreased IFNβ-induced pSTAT2 levels that were sustained for the duration of the experiment (Fig 4A). Deletion of STAT2 abolished vIRF-1-promoted pSTAT1 decay (Fig 4B), again demonstrating the critical role of STAT2 in pSTAT1 regulation by vIRF-1. A model of vIRF-1 activity with respect to pSTAT1 and pSTAT2 suppression based on the presented data is shown in Fig 7.

There is precedent in HHV-8 and other viruses for the inhibitory targeting of ISGF3 or its components as a means of evasion of host-cell innate immunity. For HHV-8, the ORF10-encoded protein is able to form inhibitory complexes with IFN receptor-associated STAT2, suppressing (by undetermined specific interactions or mechanism) IFNα-induced tyrosine phosphorylation (activation) of JAK1, TYK2, STAT1 and STAT2 and thereby blocking associated antiviral signaling [45]. Among other herpesviruses, the immediate-early IE1 proteins of the betaherpesviruses human cytomegalovirus (HCMV) and human herpesvirus 6B (HHV-6B) are able to bind to STAT2 and inhibit promoter binding by ISGF3 [46,47], and the M27 protein (pM27) of murine CMV (MCMV) targets and destabilizes STAT2 [48]. In the case of HCMV IE1, its targeting of STAT2 was found not to be associated with inhibition of STAT2, or STAT1, phosphorylation and accumulation of pSTAT1/2 in response to IFN-I stimulation, but rather with inhibition of ISGF3 via nuclear sequestration of the complex away from cognate promoters. The nuclear colocalization of IE1 and STAT2, inhibition of IFN-I signaling, and promotion of virus replication by IE1, were dependent on interaction of the viral and cellular proteins [47]. Similarly, HHV-6B IE1 interaction with STAT2 was found to correlate with suppression of IFN-I-mediated induction of ISGF3-responsive genes; introduction of the HHV-6B-specific STAT2-binding region of IE1 to HHV-6A IE1 conferred this suppressive activity to the HHV-6A protein [49]. For MCMV pM27, the destabilization of STAT2 was found to occur independently of STAT1, and STAT1 activation and associated gene induction were equivalent in wild-type and M27-null virus-infected cells, either STAT2-positive (wild-type) or STAT2-negative [48]. Examples of ISGF3 targeting and inhibition by other viruses include porcine kobuvirus non-structural VP3-protein interaction with both STAT2 and IRF9, preventing IFNβ-induced ISGF3 complex formation and also STAT2 dimerization [50], measles virus V protein and phlebovirus non-structural protein (NSP) associations with ISGF3 components, inhibiting their nuclear localization [51–53], and porcine coronavirus NSP5 protease cleavage of STAT2 [54]. The effects of particular viral proteins on STAT1 and STAT2 tyrosine phosphorylation differed, with measles V protein having no effect on IFN-I-induced tyrosine phosphorylation of either STAT and phlebovirus NSPs of Heartland virus (HRTV) and severe fever with thrombocytopenia syndrome virus (SFTSV) showing inhibitory activity with respect to phosphorylation of STAT2, specifically. Interestingly, SFTSV, but not HRTV, NSPs were able to block the nuclear localization of STAT1 in addition to STAT2. In contrast to these findings in other viral systems, we have identified suppression by vIRF-1 of tyrosine phosphorylation of both STAT1 and STAT2, STAT2-dependent suppression of pSTAT1 by vIRF-1, vIRF-1 inhibition of TYK2 activity, and vIRF-1 suppression of TYK2 interactions with STAT2 and IFNAR1; thus, vIRF-1 acts at multiple levels in the signaling pathway, directly targeting ISGF3 components and inhibiting TYK2, to effect suppression of IFN-I signaling. It is notable that while HHV-8 ORF10-protein appears to blunt STAT1, STAT2 and JAK activation and IFN-I signaling via STAT2/IFNAR-associated inhibitory complexing [45], vIRF-1 mediates these effects via inhibition and/or disruption of TYK2-IFNAR, TYK2-STAT2, and ISGF3-protein interactions, in addition to TYK2 inactivation. The use of distinct mechanisms of IFN-I suppression by different HHV-8 proteins, in addition to multi-level regulation of IFN-I signaling by vIRF-1, is likely to achieve effective inhibition of IFN-I-mediated host-cell defense and enable efficient virus replication. Determining the contributions of vIRF-1 interactions with STAT1, STAT2, IRF9 and TYK2 in virus biology will require their molecular dissection and genetic (or alternative) disruption in phenotypic studies, and this is being pursued.

In addition to investigations in transfected cells of vIRF-1 interactions with STAT1, STAT2, and TYK2 and corresponding inhibitory activities with respect to phosphorylation and downstream STAT1/2 complexing and ISGF3-associated signaling, we have also identified related effects of vIRF-1 in the contexts of infected PEL and iSLK cells. In the former, vIRF-1 depletion in iBCBL-1 cells led to markedly increased pSTAT1 levels in both uninduced (latently infected) and Dox-induced, lytically reactivated cultures (Fig 2A). Although we could not detect pSTAT2 in either phase in untreated cells, IFNβ-activated pSTAT2, along with pSTAT1, was detectable and was increased in the vIRF-1-depleted cells, both latently and lytically infected. Indeed, the detection of effects of vIRF-1 depletion on pSTAT1 and pSTAT2 in cells treated with IFNβ provides evidence of their regulation independently of any contribution of vIRF-1-suppressed IFN-I protein expression. In the context of lytically-reactivated HHV-8 BAC16-infected iSLK cells, vIRF-1 knockout led to detectably increased levels of pSTAT1 in lytically infected cells in the absence and presence of IFNβ and of pSTAT2 in the latter (Fig 2B). Thus, while it has been demonstrated that vIRF-1 ablation leads to increased IFN-I production during productive replication in iSLK cells [21], the detection of vIRF-1 suppression of pSTAT1 and pSTAT2 in the presence of exogenously added IFNβ demonstrates inhibition specifically of IFNAR-mediated signaling. Reflecting pSTAT1/2 regulation by vIRF-1 in lytically infected iBCBL-1 and iSLK cells, vIRF-1 depletion or ablation led to ISG induction in these cells, in the absence and presence of IFNβ (Fig 2D and 2E). Although our data do not correlate these effects of vIRF-1 with STAT1/2, IRF9 or TYK2 interactions, they do demonstrate inhibitory activities of virus-expressed vIRF-1 on IFN signaling components and IFNβ activity and are consistent with the interactions and effects detected in transfected cells. Furthermore, the present functional and mechanistic studies of vIRF-1 targeting and inhibition of ISGF3 proteins and TYK2 and related IFN-I-signaling inhibition are generally consistent with our and others’ previously reported phenotypic studies of vIRF-1, in which vIRF-1 depletion or ablation in PEL and iSLK cells led to reduced virus production or lytic gene expression [9,11,19,21,24], although a genetics-based study similar to our own [9] did not detect effects of vIRF-1 ORF disruption on HHV-8 replication in iSLK cells [55]. While the reported pro-replication effects of vIRF-1 are likely to involve multiple mechanisms, including suppression of IFN-I induction [6,19,21], our data show that in lytically-reactivated iBCBL-1 and iSLK cells, vIRF-1 can inhibit IFNβ signaling directly via suppression of IFN-I-activated pSTAT1 and pSTAT2, and such suppression occurred also in latently-infected iBCBL-1 cells.

In summary, the data presented here identify the first examples of vIRF-STAT and vIRF- IRF9 interactions, vIRF-1 inhibitory interaction with TYK2, and the suppression of active pSTAT1, pSTAT2, and associated ISGF3 complexing by vIRF-1. These data reveal novel means of HHV-8 interference with innate immune signaling and vIRF-1-specific and multifaceted mechanisms of suppression of pSTAT1/2 and ISGF3 activity. The identified interactions could potentially be targeted for inhibition to release virus suppression of host-cell antiviral signaling as a means of inhibiting virus replication and possibly treating HHV-8-associated cancers.

## Materials and methods

### Plasmids

Lentiviral vector-based eukaryotic expression plasmids for StrepII+Flag (SF)-tagged vIRF-1 and vIRF-2 and chitin-binding domain (CBD)-fused vIRF-1 have been reported previously [9,10]. Lentiviral vectors expressing vIRF-1 mRNA-directed shRNA (vIRF-1 shRNA-3) or non-silencing (NS) control shRNA have been described [9]. Lentiviral plasmid vectors expressing CBD-tagged STAT1, STAT2, IRF9 and IFNAR1 were generated by replacing RFP coding sequences in pCEBZ-RFP-CBD [56] with NotI/BamHI-, XhoI- or BsmBI-flanked sequences encoding the respective proteins, PCR amplified from a cDNA library (HeLa, Clontech, catalog number 638862) using appropriate custom primers. Similarly, pCEBZ-based expression vectors for S-peptide-tagged vIRF-1, JAK1 and TYK2 were generated by PCR amplification of the respective sequences, from plasmid templates (including pDONR223-JAK1, Addgene #23932 and pDONR223-TYK2, Addgene #23908, deposited by Drs. W. Hahn and D. Root), with NotI/BamHI-, XhoI- or BsmBI-flanked PCR primers and replacement of RFP coding sequences in the previously reported vector pCEBZ-RFP-S [56]. Lentiviral plasmid vectors pCEBZ-STAT1-SF and pCEBZ-IRF9-SF were generated by insertion of PCR-generated, NotI- and BamHI-flanked coding sequences into the corresponding cloning sites of pCEBZ-VKORC1v2-SF [57], replacing VKORC1v2 sequences. For prokaryotic expression of Flag-tagged STAT1, STAT2 and IRF9, pET-Flag-STAT1-His_6_, pET-Flag-STAT2-His_6_ and pET-Flag-IRF9-His_6_ vectors were generated by insertion of PCR-generated, NdeI- and SalI-flanked coding sequences into the corresponding cloning sites of pET22b(+) (Novagen, catalog number 69744). The vIRF-1 ORF fused with S-peptide coding sequence was cloned between the NdeI and XhoI sites of pET22b(+) to generate vector pET-S-vIRF-1-His_6_ for prokaryotic expression of S-tagged vIRF-1. GST-fused vIRF-1 was expressed from the prokaryotic vector pGEX-vIRF-1 [24]. IFNβ-responsive reporter plasmid pGL3-ISRE-Luc was kindly provided by Dr. Shaoli Lin.

### Cell culture and transfection

TRExBCBL1-RTA cells [35] were grown in RPMI 1640 medium supplemented with 10% FBS and 10 μg/ml gentamicin. HEK293T (293T) and iSLK [36] cells were cultured in Dulbecco’s modified Eagle’s medium (DMEM) supplemented with 10% fetal bovine serum (FBS) and 10 μg/ml gentamicin. For routine transfections, 293T cells at 40–60% confluence were transfected with mixtures of plasmid DNA and cationic polymer linear polyethylenimine (Polysciences, catalog number 593215), as previously reported [58].

### Generation of STAT1, STAT2, and IRF9 knockout cell lines

CRISPR vector lentiCRISPRv2-Blast [59], obtained from Addgene (#98293, deposited by Dr. Brett Stringer), was used for transduction of 293T cells and coexpression of Cas9 and gene-specific gRNA. For the latter, double-stranded (ds) oligonucleotides containing target gene sequences flanked by BsmBI restriction sites were cloned between the BsmBI sites of lentiCRISPRv2-Blast. Guide RNA sequences, including lentiviral vector cloning-compatible flanking nucleotides (upper case), were as follows: STAT1-gRNA, Forward: CACCGcatggaaatcagacagtacc, Reverse: AAACggtactgtctgatttccatgC; STAT2-gRNA, Forward: CACCGgaatcttgacagcccctttc, Reverse: AAACgaaaggggctgtcaagattcC; IRF9-gRNA, Forward: CACCGgaactgggtggtggagcaag, Reverse: AAACcttgctccaccacccagttcC. The respective lentiviruses, produced in transfected 293T cells (see below), were used to infect 293T cultures in the presence of 5 μg/ml of polybrene. The infected cells, cultured in DMEM supplemented with 10% FBS, were treated with blasticidin (10 μg/ml) for two weeks to select for lentivirus-positive cells; clonal cell lines were derived by limiting dilution, culturing in 96-well plates, and then a subset of these lines were selected for further expansion and analyzed by immunoblotting for the absence of the relevant target-gene product.

### Lentivirus production

For routine lentivirus production, 75-cm^2^ flasks containing ~80%-confluent 293T cultures were cotransfected with 12 μg of lentiviral vector DNA and 9 μg and 3 μg, respectively, of psPAX2 and pMD2.G packaging plasmids (Addgene #12260 and #12259, deposited by Dr. Didier Trono). After 6 h, the inoculum was replaced with fresh medium (DMEM containing 10% FBS) and the cells were cultured for a further 48 h. Lentivirus-containing medium was harvested and lentiviruses were pelleted by ultracentrifugation at 25,000 rpm in a SW32Ti rotor for 2 h at 4°C; virus pellets were resuspended in 5 ml of RPMI 1640 medium supplemented with 10% FBS to produce lentivirus stocks, stored at -80°C. For lentivirus production for CRISPR-mediated gene ablation, 8 μg, 6 μg, and 2 μg of CRISPRv2-Blast, psPAX2, and pDM2.G vectors, respectively, were cotransfected into ~80%-confluent 293T cultures in 10-cm dishes. Following post-transfection medium replacement and incubation for two days in fresh medium, the virus-containing culture medium was harvested and filtered through 0.45 μm membranes; the cleared virus preparation was used to inoculate fresh 293T cultures for generation of gene-ablated cell lines (see above).

### Immuno- and affinity-precipitations

For immuno- and affinity-precipitations of Flag-, CBD-, and S-tagged proteins, 293T cells were cotransfected with the appropriate “bait” and “prey” expression vectors. Cells were harvested 48 h posttransfection and lysed in NP40 lysis buffer (50 mM Tris-HCl [pH7.5], 150 mM NaCl, 5 mM EDTA, 0.2% NP40) containing protease inhibitor cocktail (Sigma, catalog number P8340) for 1 hour at 4°C and clarified by centrifugation at 16,000 x g for 10 min at 4°C. The supernatants were used in the subsequent coprecipitation assays according to the manufacturer’s instructions for Flag-antibody beads (Sigma, catalog number M8823), chitin resin (New England Biolabs, S6651) or S-protein agarose (Novagen, 69704). Incubations were carried out at 4°C overnight, with gentle shaking. Bound precipitates were washed three-to-five times with NP40-containing wash buffer (50 mM Tris-HCl [pH 7.5], 150 mM NaCl, 0.05% NP40). Coprecipitates were released by adding SDS-PAGE loading buffer and boiling at 95°C for 10 min. prior to gel fractionation and immunoblotting.

For *in vitro* coprecipitation and binding-competition assays, BL21(DE3) cells transformed with pET22b(+)- or pGEX-4T-1-based vectors (see above) were used to generate His_6_- or GST-linked proteins; expression was induced by addition of 1 mM isopropyl-β-D-thiogalactopyranoside (IPTG) for 24 h. Pelleted cells were resuspended in lysis buffer (300 mM NaCl, 10 mM imidazole, 50 mM NaH_2_PO_4_ [pH 8.0]), for His_6_-tagged protein, or PBS (137 mM NaCl, 2.7 mM KCl, 10 mM Na_2_HPO_4_ [pH 7.4]), for GST fusions, containing 1 mg/ml lysozyme. After 10 min. sonication, the lysates were cleared by centrifugation at 12,000 x g for 10 min at 4°C. Then, the supernatants were incubated with either Ni-NTA (Qiagen, catalog number 30210) or glutathione sepharose (GE Healthcare, catalog number 17-0756-01) beads at 4°C for 1 h. Subsequently, the beads were washed three to five times with lysis buffer (His_6_ precipitates) or PBS (GST precipitates) prior to protein release with His_6_ elution buffer (300 mM NaCl, 250 mM imidazole, 50 mM NaH_2_PO_4_ [pH8.0]) or glutathione buffer (10 mM reduced glutathione, 50 mM Tris-HCl [pH 8.0]). Purified proteins were checked for integrity and purity by Coomassie Brilliant Blue or Ponceau S staining of SDS-polyacrylamide gel-fractionated proteins. *In vitro* coprecipitation assays employed S-peptide and His_6_ dually-tagged vIRF-1 and Flag- and His_6_-tagged STAT1, STAT2 and IRF9; paired proteins were incubated on ice for 2 h in Tris-buffered saline, TBS (50mM Tris-HCl [pH7.5], 150mM NaCl), containing S-protein agarose. Following incubation, the beads were washed by repeated precipitation and resuspension in ice-cold TBS. The beads were boiled in SDS-PAGE loading buffer (10 min.), prior to SDS-PAGE fractionation and immunoblotting of released proteins.

For immunoprecipitations of endogenous proteins, iBCBL-1 cells were pelleted and washed in PBS prior to disruption in lysis buffer (50 mM Tris-HCl [pH 7.5], 150 mM NaCl, 5 mM EDTA, 0.2% NP40) containing protease inhibitor cocktail (Sigma, P8340) and sonication using a micro-tip. After centrifugation at 14,000 × g for 15 min at 4°C, samples of the cleared lysates were incubated with primary antibodies (to IRF9, STAT1 or STAT2) overnight at 4°C. Protein A/G magnetic beads (Thermo Scientific, 88803) were then added, and incubation continued at 4°C for 2 hours. The beads and associated proteins were washed three to five times with wash buffer (50 mM Tris-HCl [pH 7.5], 150 mM NaCl and 0.2% NP40). Co-immunoprecipitates were eluted using IgG elution buffer (Thermo Scientific, 21028) and denatured in SDS-PAGE loading buffer by boiling in 95°C for 10 min. prior to gel fractionation and immunoblotting.

### Proximity ligation assays

Assays were carried out using the mouse/rabbit Duo-link In Situ Red Kit from Millipore-Sigma-Aldrich (catalog number DUO92101), according to the manufacturer’s instructions. Briefly, iBCBL-1 cells seeded on 8-well glass chamber slides and fixed in PBS containing 4% paraformaldehyde (15 min., room temperature) were blocked in kit-supplied blocking solution (37°C for 1 hour) prior to incubation with primary antibodies in kit-supplied antibody buffer (4°C overnight), PLA probe solution (37°C, 1 hour), ligation solution (37°C, 30 minutes), and amplification solution (37°C, 100 min.), and rinsing with 1x and then 0.01x manufacturer-supplied wash buffer. The slides were then mounted with a coverslip using a minimal volume of kit-supplied mounting solution containing DAPI. The PLA signals were observed and photographed using a Leica SP8 confocal microscope.

### Antibodies

Primary antibodies used for immunoblotting were: S (Abcam, catalog number ab184223); CBD (New England BioLabs, E8034S); Flag (Sigma, F1804); β-actin (Sigma, A5316); GAPDH (Invitrogen, TAB1001); vIRF-1 (rabbit polyclonal antiserum, provided by Dr. Gary Hayward); STAT1, pSTAT1, IRF9, His_6_, GST and p53 from Santa Cruz Biotechnologies (catalog numbers sc464, sc-365893, sc-8394, sc-803, sc-138, and sc-126, respectively); STAT2 and pSTAT2 from Cell Signaling Technology (catalog numbers 72604 and 4441). Horse radish peroxidase (HRP)-conjugated secondary antibodies were: anti-rabbit IgG (Cell Signaling Technology, 7074; Rockland, 18-8816-33); anti-mouse IgG (Cell Signaling Technology, 7076; Rockland, 18-8817-33). For immunoprecipitations, IRF9 antibodies from BioLegend (catalog number 660702) and Santa Cruz Biotechnologies (sc-365893) and STAT1 and STAT2 antibodies from Santa Cruz Biotechnologies (sc-464 and sc-1668) were used. Rat and mouse antibodies against human STAT1 (Biotechne, MAB1490) and STAT2 (Santa Cruz Biotechnologies, sc-514193) were used along with vIRF-1-directed rabbit antiserum (see above) in PLA.

### Luciferase reporter assays

For ISRE-luciferase assays, ~50%-confluent 293T cells in 12-well tissue culture plates were PEI-cotransfected with 0.05 μg pGL3-ISRE-Luc, 0.005 μg pRL-TK (Renilla luciferase normalization control), and 0.5 μg of vIRF-1 and/or TYK2 expression vector, with or without IFNβ treatment. After 24 h, cells were lysed with lysis buffer and analyzed sequentially for firefly and Renilla luciferase activity using commercial reagent (Promega, catalog number E2920) according to the manufacturer’s instruction. Relative firefly luciferase activities were calculated after normalization to Renilla luciferase activity, to account for any differences in transfection efficiencies between cultures.

### RT-qPCR analysis of gene expression

RNA isolation and reverse transcription and was carried out essentially as reported previously [10]. Briefly, TRIzol reagent (Invitrogen, catalog number 15596026) was used for RNA isolation, according the manufacturer’s protocol, and RNA was treated with DNase I prior to cDNA synthesis with Superscript VI (Invitrogen, catalog number 18090010). First-strand cDNA was then used for PCR using SYBR green PCR master mix (Thermo Fisher Scientific, cat. no. 4367659), as described previously [24]. PCR primers were: IFNβ(F), CAGTCCTGGAAGAAAAAC; IFNβ(R), TCAGTTTCGGAGGTAACCTGT; ISG15(F), CACCGTGTTCATGAATCTGC; ISG15(R), CTTTATTTCCGGCCCTTGAT; ISG56(F), CCTCCTTGGGTTCGTCTACA; ISG56(R), GGCTGATATCTGGGTGCCTA; vIRF-1(F), CCGGACACGACAACTAAGAA; vIRF-1(R), GTCTCTGCGCCATTCAAAAC; GAPDH(F), CCAGGTGGTCTCCTCTGACTTCTC, GAPDH(R), ATACCAGGAAATGAGCTTGACA. Target-transcript levels between samples were normalized to GAPDH mRNA levels for quantitative comparisons.

## Supporting information

S1 Fig Identification of vIRF-1 interactions with ISGF3 components. (A) Testing for vIRF-1 interaction with STAT1. 293T cells were cotransfected with expression vectors for Flag-tagged vIRF-1 (virf1), vIRF-2 (virf2, for comparison), or empty vector (-) negative control and chitin-binding domain (CBD)-fused STAT1. The vIRFs were sedimented from cell lysates by Flag-immunoprecipitation (IP) and coprecipitated STAT1 was identified by CBD immunoblotting. Precipitation of each vIRF and the presence of STAT1-CBD in each transfectant was confirmed by Flag and CBD immunoblotting of immunoprecipitates and cell lysates, respectively. The dotted line indicates deletion of a lane. (B) Reciprocal affinity-precipitation (AP) of STAT1-CBD and immunoblot detection of coprecipitated vIRF-1-Flag. (C-D) Transfection-based coprecipitation experiments equivalent to those of panels A and B were performed using CBD-fused STAT2. For CBD-AP (D), vIRFs 1 and 2 were coexpressed in the same transfectants and the proteins were distinguished on Flag immunoblots by their different sizes. Dotted lines indicate deletions of lanes. (E) Affinity-precipitation assay for association of Flag-tagged IRF9 and S-peptide-tagged vIRF-1 (vIRF-1-S, protein-S-precipitated), expressed in vector-transfected 292T cells. Empty vector (-) was used as a negative control. (F) A similar experiment was carried out using affinity precipitation of IRF9-CBD and detection of coprecipitated Flag-tagged vIRF-1.
(TIF)

S1 Chart Data Compiled primary and processed data underlying charts presented in the manuscript. (XLSX)
