# Supplementary figures and images for: STAT and Janus kinase targeting by human herpesvirus 8 interferon regulatory factor in the suppression of type-I interferon signaling
Source: PLoS Pathog. 2022 Jul 1;18(7):e1010676. doi: 10.1371/journal.ppat.1010676 (PMC9307175; doi:10.1371/journal.ppat.1010676)

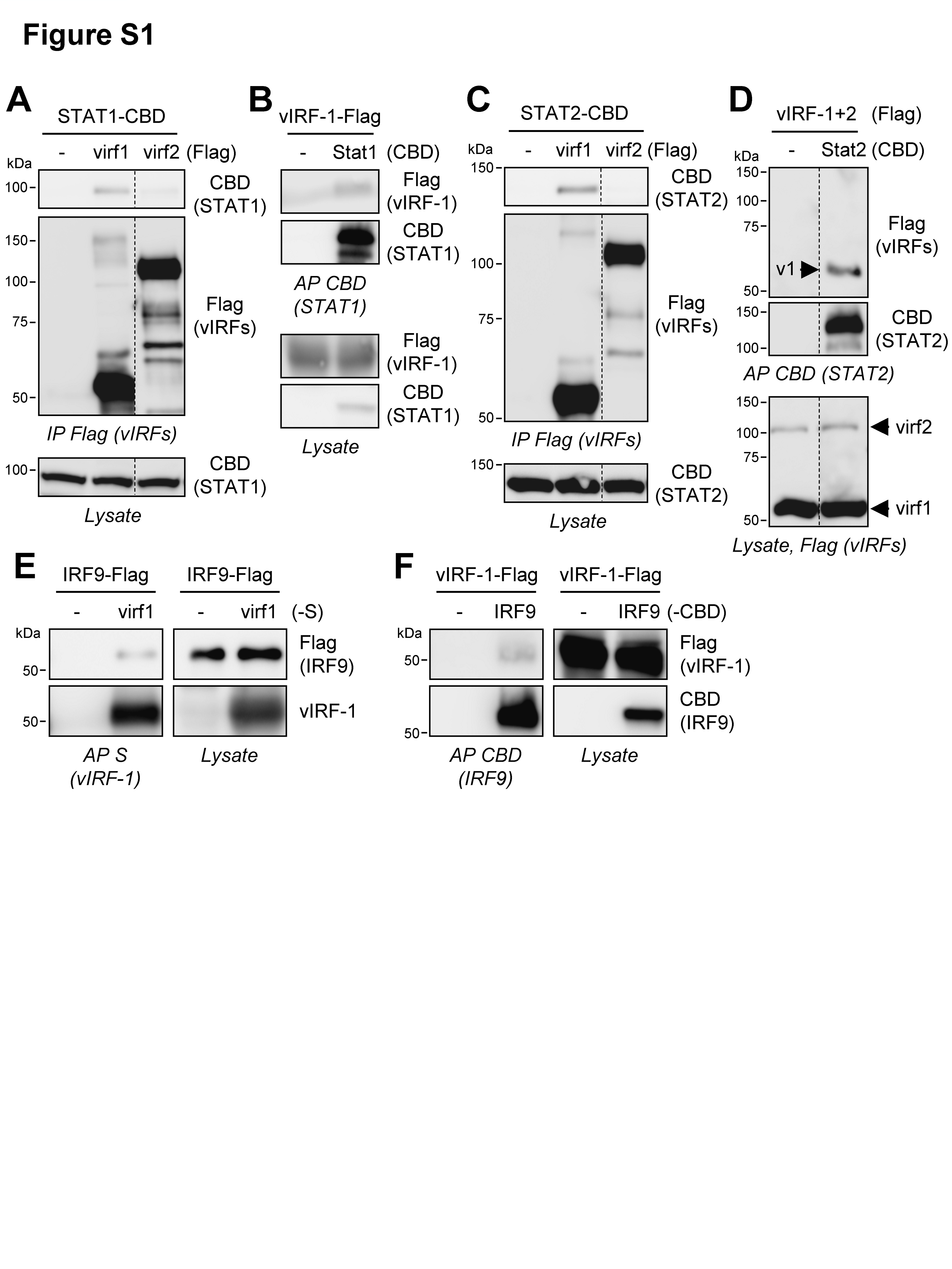

Supplement: S1 Fig — (A) Testing for vIRF-1 interaction with STAT1. 293T cells were cotransfected with expression vectors for Flag-tagged vIRF-1 (virf1), vIRF-2 (virf2, for comparison), or empty vector (-) negative control and chitin-binding domain (CBD)-fused STAT1. The vIRFs were sedimented from cell lysates by Flag-immunoprecipitation (IP) and coprecipitated STAT1 was identified by CBD immunoblotting. Precipitation of each vIRF and the presence of STAT1-CBD in each transfectant was confirmed by Flag and CBD immunoblotting of immunoprecipitates and cell lysates, respectively. The dotted line indicates deletion of a lane. (B) Reciprocal affinity-precipitation (AP) of STAT1-CBD and immunoblot detection of coprecipitated vIRF-1-Flag. (C-D) Transfection-based coprecipitation experiments equivalent to those of panels A and B were performed using CBD-fused STAT2. For CBD-AP (D), vIRFs 1 and 2 were coexpressed in the same transfectants and the proteins were distinguished on Flag immunoblots by their different sizes. Dotted lines indicate deletions of lanes. (E) Affinity-precipitation assay for association of Flag-tagged IRF9 and S-peptide-tagged vIRF-1 (vIRF-1-S, protein-S-precipitated), expressed in vector-transfected 292T cells. Empty vector (-) was used as a negative control. (F) A similar experiment was carried out using affinity precipitation of IRF9-CBD and detection of coprecipitated Flag-tagged vIRF-1. (TIF) [file ppat.1010676.s001.tif]
